# Supplementary material for: Memory specificity is linked to repetition effects in event-related potentials across the lifespan
Source: Dev Cogn Neurosci. 2021 Jan 27;48:100926. doi: 10.1016/j.dcn.2021.100926 (PMC7868631; doi:10.1016/j.dcn.2021.100926)
Supplement: Supplementary file 1 [file mmc1.docx]

# Supplemental material

**Encoding conditions**

The 80 object categories were equally divided into four conditions (see Figure S1) that differed with respect to (a) the number of different presented exemplars from one category (either two or four), and (b) the number of exemplar repetitions (either two or four times). This resulted in four encoding conditions: (1) Baseline (BL) condition with two exemplars per category that were each presented twice, (2) High-Repetition (HR) condition with two exemplars per category that were each presented four times, (3) High-Exemplar (HE) condition with four exemplars per category that were each presented twice, (4) High-Repetition-and-Exemplar (HRE) condition with four exemplars per category that were each presented four times.

Twenty object categories were randomly selected for each condition. For those conditions with two exemplars per category (BL and HR), two of the four available exemplars were randomly selected for presentation. Since the number of exemplars and repetitions differed between conditions, the number of trials in each condition varied accordingly: 80 in the BL condition, 160 in the HR condition, 160 in the HE condition, and 320 trials in the HRE condition. This adds up to a total of 720 trials. A shorter version of the task was used for the children. They performed only the first half of the adult task, comprising 40 distinct object categories, i.e., 10 in each encoding condition, which results in 360 experimental trials (40 BL, 80 HR, 80 HE, 160 HRE).

For those categories that belonged to an encoding condition with four exemplars (HE and HRE), two of these were randomly selected to be tested in the recognition task (as old items).

Since we did not have specific hypotheses how these encoding conditions would affect event-related repetition effects, for the purpose of the analysis presented in this article, we collapsed across all conditions and focused on the comparison of the first and second presentation of an object, independent of its condition.

**
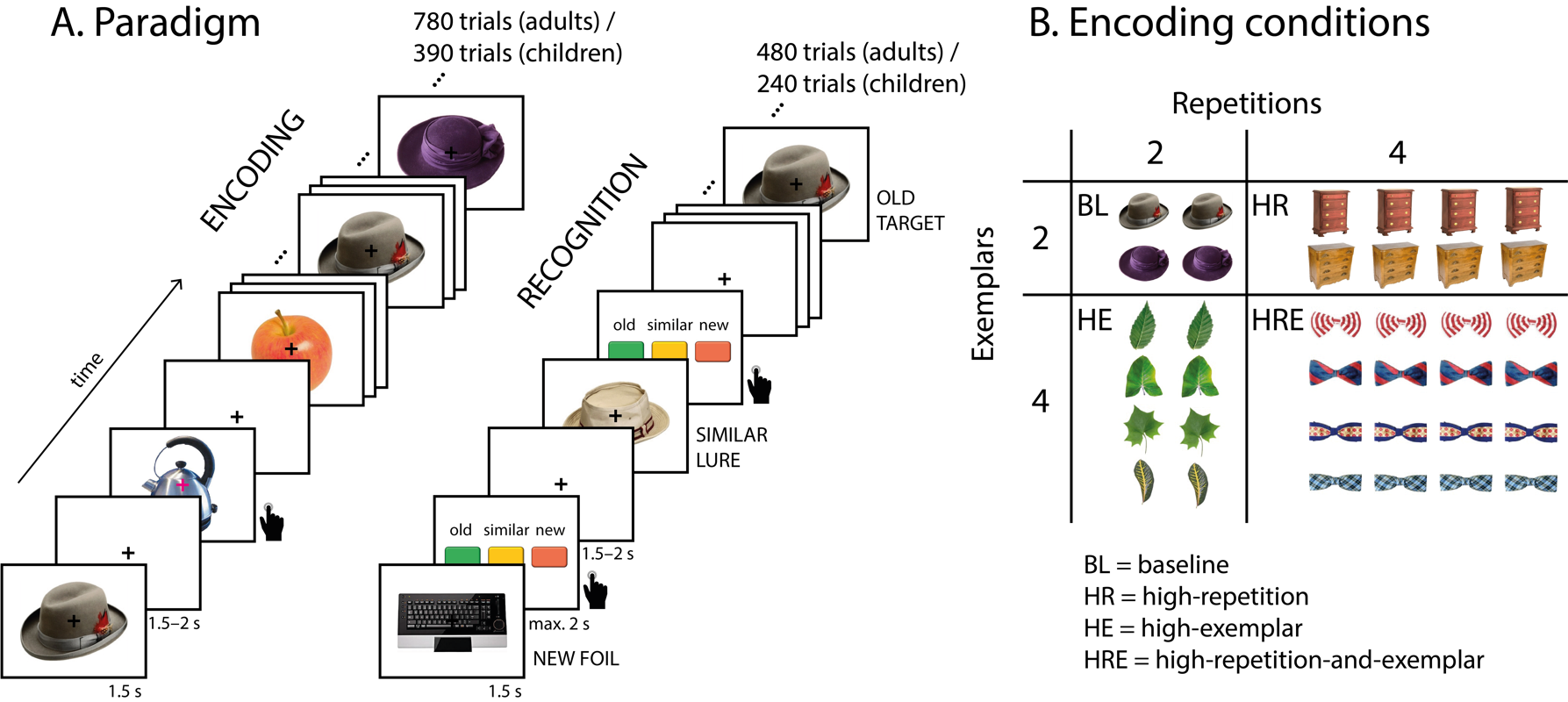
**

**Figure S1.** Encoding conditions. The number of item repetitions (2 or 4) and category exemplars (2 or 4) were manipulated during encoding, resulting in 4 encoding conditions: Baseline (BL), High-Repetition (HR), High-Exemplar (HE), and High-Repetition-and-Exemplar (HRE). Each condition comprised 20 object categories for adults, and 10 object categories for children. One sample category per condition is shown for illustration.

**The effect of repetitions on item memory**

In the following, we asked whether item and category repetitions might affect memory performance differentially in children, younger adults, and older adults. We initially expected better item-specific memory performance when items were repeated more often and worse performance when more exemplars were presented during encoding. This was based on the hypothesis that more repetitions would provide more opportunity to encode item-specific details leading to both better target recognition as well as rejection of similar lures (Benjamin, 2001; but see Reagh and Yassa, 2014), whereas more exemplars would create stronger interference, making it harder to distinguish between similar items (Anderson, 2015). The latter is supported by previous findings showing that increasing the number of exemplars from the same object category, reduced participants’ ability to discriminate between lures and targets from that category (Gallo, 2004; Konkle et al., 2010; Omohundro, 1981; Poch et al., 2019). Alternatively, interference from similar memories can also trigger a repulsion of memory representations, suggested by the findings of less overlapping activation patterns in the hippocampus (pattern separation), which was associated with less memory interference, i.e., better performance (Chanales et al., 2020, 2017; Favila et al., 2016). That is, both scenarios – better or worse item memory when more exemplars were presented – are conceivable.

To examine these predictions, we conducted a three-way mixed ANOVA with age group as the between-subjects factor, number of repetitions and number of exemplars as within-subjects factors, and item memory performance as the dependent variable. The results revealed a main effect of age (*F*(2,109) = 7.48, *p* < 0.001) with the same pattern of age differences as reported in the main paper, and a main effect of the number of exemplars (*F*(1,109) = 8.53, *p* = 0.004). Post-hoc *t*-tests demonstrated better item memory when four rather than two exemplars were presented (*t*(111) = 3.58, *p* < 0.001), revealing a beneficial effect of a higher number of category exemplars. This is in line with the repulsion prediction (see above), whereas the number of repetitions did not influence item memory (*F*(1,109) = 0.99, *p* = 0.321). To better disentangle the effect of repetitions and exemplars for item-specific memory, we further examined the potential effect of repetitions by directly contrasting memory performance for items from the HR and BL condition, i.e., independently of the exemplar manipulation. Across age groups, this revealed better item memory for the HR condition (*t*(111) = 2.72, *p* = 0.008; paired-sample *t*-test).

Since there were no interactions between age and item manipulations, we largely disregard the encoding conditions and do not separate the trials accordingly in the analyses presented here.

## Lifespan differences in ERPs

The ERPs of children, young adults, and older adults at selected representative electrode sites are shown in Figure S2. Children exhibited overall higher amplitudes than adults, which is consistent with previous findings in the literature (Coch et al., 2005; Dustman and Beck, 1969; Mueller et al., 2008). This could, for example, be due to differences in skull thickness (Frodl et al., 2001) but in the current study it may also be a result of the different EEG systems and laboratories used to test the children and adults.

**Figure S2**. Event-related potentials (ERPs) at selected electrode sites, averaged over all trials in which an object was shown for the first time, for all children (blue), young adults (black), and older adults (purple). The *x*-axis shows trial time (s) with stimulus onset at 0 (origin) and offset at 1.5 s, the *y*-axis shows amplitude (*µ*V) with negative values plotted downwards. Data were baseline-corrected with an absolute pre-stimulus baseline of 200 ms.

**Repetition suppression for all presentations**


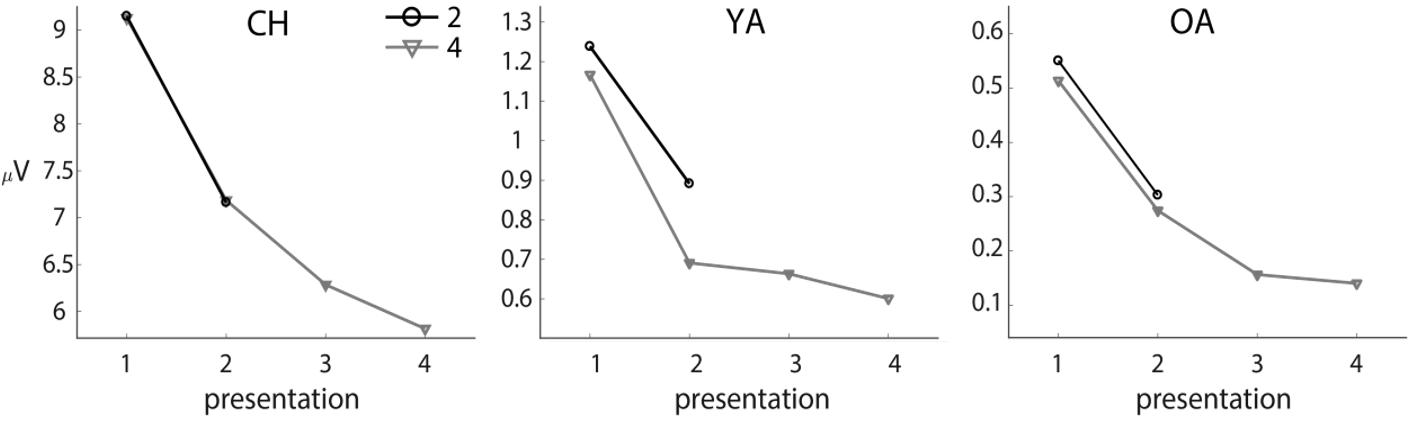


**Figure S3.** Mean amplitudes (µV; y-axis) within identified clusters (first repetition suppression effect) for all repeated presentations (x-axis), averaged over conditions with two repetitions (N and HE; black line) and conditions with four repetitions (HR and HRE, gray line) for children (CH; left), young adults (YA; center), and older adults (OA; right).

**Table S1.** **Covariates**

| Group | Processing speed DSST^1^ *M*(*SD*) | Verbal knowledge STW^2^ *M*(*SD*) | Dementia screening MMSE^3^ *M*(*SD*) | Verbal digit span task^4^ *M*(*SD*) |
| --- | --- | --- | --- | --- |
| Children | 29.62(7.00) | N/A | N/A | 13.43(2.04) |
| Young adults (EEG) | 63.97(10.67) | 22.21(4.16) | N/A | N/A |
| Young adults (control) | 70.2(6.3)* | N/A | N/A | 20.83(3.06) |
| Older adults | 47.58(8.99) | 28.72(3.30) | 28.95(1.21) | N/A |
| *M = mean, SD = standard deviation, ^1^ Digit Symbol Substitution Test (Wechsler, 1981): number of correct responses in 90 s, * for the control young adults group, test time was mistakenly set to 120 s; the provided value is converted to responses / 90 s, ^2^ Spot-the-Word test (Mehrfachwahl-Wortschatz-Intelligenztest; Lehrl, 1977): number of correct responses, ^3^ Mini-Mental State Examination (Folstein et al., 1975); ^4^ Petermann and Petermann, 2011* | | | | |

**References**

Anderson, M.C., 2015. Incidental forgetting, in: Baddeley, A., Eysenck, M.W., Anderson, M.C. (Eds.), Memory. Psychology Press, New York, NY, pp. 231–264.

Benjamin, A.S., 2001. On the dual effects of repetition on false recognition. J. Exp. Psychol. Learn. Mem. Cogn. 27, 941–947. https://doi.org/10.1037/0278-7393.27.4.941

Chanales, A.J.H., Oza, A., Favila, S.E., Kuhl Correspondence, B.A., Edu, A.C., Kuhl, B.A., 2017. Overlap among Spatial Memories Triggers Repulsion of Hippocampal Representations. Curr. Biol. 27, 1–11. https://doi.org/10.1016/j.cub.2017.06.057

Chanales, A.J.H., Tremblay-McGaw, A.G., Kuhl, B.A., 2020. Adaptive repulsion of long-term memory representations is triggered by event similarity. bioRxiv 2020.01.14.900381. https://doi.org/10.1101/2020.01.14.900381

Coch, D., Skendzel, W., Neville, H.J., 2005. Auditory and visual refractory period effects in children and adults: an ERP study. Clin. Neurophysiol. Off. J. Int. Fed. Clin. Neurophysiol. 116, 2184–2203. https://doi.org/10.1016/j.clinph.2005.06.005

Dustman, R.E., Beck, E.C., 1969. The effects of maturation and aging on the wave form of visually evoked potentials. Electroencephalogr. Clin. Neurophysiol. 26, 2–11. https://doi.org/10.1016/0013-4694(69)90028-5

Favila, S.E., Chanales, A.J.H., Kuhl, B.A., 2016. Experience-dependent hippocampal pattern differentiation prevents interference during subsequent learning. Nat. Commun. 6, 1–10. https://doi.org/10.1038/ncomms11066

Folstein, M.F., Folstein, S.E., McHugh, P.R., 1975. “Mini-mental state”: A practical method for grading the cognitive state of patients for the clinician. J. Psychiatr. Res. 12, 189–198. https://doi.org/10.1016/0022-3956(75)90026-6

Frodl, T., Meisenzahl, E.M., Müller, D., Leinsinger, G., Juckel, G., Hahn, K., Möller, H.J., Hegerl, U., 2001. The effect of the skull on event-related P300. Clin. Neurophysiol. Off. J. Int. Fed. Clin. Neurophysiol. 112, 1773–1776.

Gallo, D.A., 2004. Using Recall to Reduce False Recognition: Diagnostic and Disqualifying Monitoring. J. Exp. Psychol. Learn. Mem. Cogn. 30, 120–128. https://doi.org/10.1037/0278-7393.30.1.120

Konkle, T., Brady, T.F., Alvarez, G.A., Oliva, A., 2010. Conceptual distinctiveness supports detailed visual long-term memory for real-world objects. J. Exp. Psychol. Gen. 139, 558–578. https://doi.org/10.1037/a0019165

Lehrl, S., 1977. Mehrfachwahl-Wortschatz-Intelligenztest MWT-B. Straube, Erlangen.

Mueller, V., Brehmer, Y., von Oertzen, T., Li, S.-C., Lindenberger, U., 2008. Electrophysiological correlates of selective attention: A lifespan comparison. BMC Neurosci. 9, 18. https://doi.org/10.1186/1471-2202-9-18

Omohundro, J., 1981. Recognition vs. classification of ill-defined category exemplars. Mem. Cognit. 9, 324–331. https://doi.org/10.3758/BF03196966

Petermann, F., Petermann, U., 2011. Wechsler intelligence scale for children - fourth edition, 2., ergänzte Auflage. ed. Pearson, Frankfurt/M.

Poch, C., Prieto, A., Hinojosa, J.A., Campo, P., 2019. The impact of increasing similar interfering experiences on mnemonic discrimination: Electrophysiological evidence. Cogn. Neurosci. 10, 129–138. https://doi.org/10.1080/17588928.2019.1571484

Reagh, Z.M., Yassa, M.A., 2014. Repetition strengthens target recognition but impairs similar lure discrimination: evidence for trace competition. Learn. Mem. 21, 342–346. https://doi.org/10.1101/lm.034546.114

Wechsler, D., 1981. WAIS-R: manual : Wechsler adult intelligence scale--revised. Harcourt Brace Jovanovich [for] Psychological Corp., New York, NY.
